# Synthesis and high temperature thermoelectric properties of Yb_0.25_Co_4_Sb_12_-(Ag_2_Te)_*x*_(Sb_2_Te_3_)_1−*x*_ nanocomposites

**DOI:** 10.3389/fchem.2015.00053

**Published:** 2015-09-03

**Authors:** Jin Zheng, Jiangying Peng, Zhexin Zheng, Menghan Zhou, Emily Thompson, Junyou Yang, Wanli Xiao

**Affiliations:** ^1^School of Mechanical Science & Engineering, Huazhong University of Science & TechnologyWuhan, China; ^2^Department of Physics and Astronomy, Clemson UniversityClemson, SC, USA; ^3^State Key Laboratory of Material Processing and Die & Mound Technology, Huazhong University of Science & TechnologyWuhan, China

**Keywords:** thermoelectric, nanocomposite, skutterudite, AgSbTe_2_, figure of merit

## Abstract

Nanocomposites are becoming a new paradigm in thermoelectric study: by incorporating nanophase(s) into a bulk matrix, a nanocomposite often exhibits unusual thermoelectric properties beyond its constituent phases. To date most nanophases are binary, while reports on ternary nanoinclusions are scarce. In this work, we conducted an exploratory study of introducing ternary (Ag_2_Te)_x_(Sb_2_Te_3_)_1−x_ inclusions in the host matrix of Yb_0.25_Co_4_Sb_12_. Yb_0.25_Co_4_Sb_12_-4wt% (Ag_2_Te)_x_(Sb_2_Te_3_)_1−x_ nanocomposites were prepared by a melting-milling-hot-pressing process. Microstructural analysis showed that poly-dispersed nanosized Ag-Sb-Te inclusions are distributed on the grain boundaries of Yb_0.25_Co_4_Sb_12_ coarse grains. Compared to the pristine nanoinclusion-free sample, the electrical conductivity, Seebeck coefficient, and thermal conductivity were optimized simultaneously upon nanocompositing, while the carrier mobility was largely remained. A maximum *ZT* of 1.3 was obtained in Yb_0.25_Co_4_Sb_12_-4wt% (Ag_2_Te)_0.42_(Sb_2_Te_3_)_0.58_ at 773 K, a ~ 40% increase compared to the pristine sample. The electron and phonon mean-free-path were estimated to help quantify the observed changes in the carrier mobility and lattice thermal conductivity.

## Introduction

In the wake of severe environmental and energy crisis, thermoelectric (TE) materials, which can convert heat to electricity directly, have attracted much attention (Dresselhaus et al., 2007; Liu et al., 2012; Alam and Ramakrishna, 2013). The conversion efficiency of a TE material is determined by its dimensionless figure of merit *ZT* = α^2^*T*/ρκ, where α, ρ, κ, *T* are Seebeck coefficient, electrical resistivity, total thermal conductivity, and absolute temperature, respectively. The term α^2^/ρ is called power factor. The total thermal conductivity κ is composed of electronic component κ_*e*_ and lattice component κ_*L*_.

A general challenge in single-phased bulk TE material is that all three TE properties, the electrical conductivity, the Seebeck coefficient, and the electronic thermal conductivity, are intimately but adversely inter-dependent, optimizing one often degrades others. In this regard, nanostructuring has provided a new route to partially decouple the TE properties, as evidenced in supperlattice (Venkatasubramanian et al., 2001; Harman et al., 2002; Zeng et al., 2009) and nanocomposites (Hsu et al., 2004; Zhao et al., 2005; Poudel et al., 2008; Fan et al., 2011; Lee et al., 2012; Liu et al., 2012; Alam and Ramakrishna, 2013), by enhancing the power factor and/or lowering the κ_*L*_. Nanostructuring in bulk materials can be realized by directly incorporating nanoparticles in the host matrix (Fan et al., 2011; Lee et al., 2012), or creating nanoparticles *in situ* (Hsu et al., 2004), or reducing the host matrix particle size down to nanometer scale (Poudel et al., 2008). A number of approaches have been proposed to enhance the power factor, mostly via enhancing the Seebeck coefficient by (i) creating sharp features in the density-of-states, for instance, by introducing resonant states (Heremans et al., 2008; Ahn et al., 2010), and (ii) increasing the energy dependence in the relaxation times, for instance, by energy filtering effect at the interface (Heremans et al., 2005; Shakouri and Zebarjadi, 2009). Recently, a modulation-doping mechanism has been proposed to enhance the mobility therefore the electrical conductivity as compared to its uniform-doping counterpart (Zebarjadi et al., 2011; Yu et al., 2012). In the mean time, many *ZT* enhancements in nanocomposites are owing to the significant reduction of κ_*L*_, via strong interface scattering of heat-carrying phonons.

CoSb_3_-based filled-skutterudites have been known as promising TE materials for medium-temperature power generation applications (Yang et al., 2007; Shi et al., 2011; Peng et al., 2012a; Rogl et al., 2014). Despite the tremendous progress in developing high performance filled-skutterudites, there is still room for further reduction of κ_*L*_, especially in *n*-type filled-skutterudites, as compared to other state-of-the-art TE materials. The future of CoSb_3_-based filled-skutterudites is largely hinged upon further reduction of κ_*L*_ while remaining or even increasing the power factor.

To this end, introducing AgSbTe_2_ nanoinclusions in the host matrix of Yb_0.2_Co_4_Sb_12_ proved to be an effective approach. AgSbTe_2_ is by itself a promising TE material (Xu et al., 2010; Ma et al., 2013). Its extremely low κ_*L*_ (~0.6 W/m-K at room temperature; Rosi et al., 1961) has been attributed to strong anharmonicity (Morelli et al., 2008; Nielsen et al., 2013), cation force-constant disorder (Ye et al., 2008), and also prominent nanostructural features including naturally formed nanoscale modulations and nanodomains with Ag and Sb ordering (Ma et al., 2013; Carlton et al., 2014). In our previous study, we chose AgSbTe_2_ as the nanoinclusion in the host matrix of micro-grained Yb_0.2_Co_4_Sb_12_ skutterudite. We found that the addition of AgSbTe_2_ nanoinclusions simultaneously optimized {ρ, α, κ} and attained much improved *ZT* values when the nanoinclusion weight percentage was between 4 and 6 wt% (Fu et al., 2013; Peng et al., 2014). Importantly, there is a topological crossover for the AgSbTe_2_ inclusions from isolated nanoparticles to nano-coating between 6 and 8 wt%. Above this crossover, the composite with high AgSbTe_2_ content turned to behave like a conventional two-phase composite that can be described by an effective-medium model, and the ρ and α were degraded (Peng et al., 2014).

Building on these results, we intend to further optimize the TE performance of filled-skutterudite nanocomposite. Note that AgSbTe_2_ can be regarded as a solid solution between Ag_2_Te and Sb_2_Te_3_ from the metallurgical viewpoint, multi-phase nanocomposites, and complicated nanostructures can be formed by varying Ag_2_Te:Sb_2_Te_3_ ratios in AgSbTe_2_-based materials (Wang et al., 2008; Zhang et al., 2010a,b). Hence, in the present work we extend the study into a more complicated phase space by varying the Ag_2_Te:Sb_2_Te_3_ ratio but fixing the overall weight percentage of (Ag_2_Te)_x_(Sb_2_Te_3_)_1−x_ at 4 wt%, in the hopes that the increased phase interfaces may enhance the α via energy filtering effect and reduce the κ_*L*_ via interface scattering.

## Experimental procedures

Yb_0.25_Co_4_Sb_12_ and (Ag_2_Te)_x_(Sb_2_Te_3_)_1−x_ (*x* = 0.36, 0.40, 0.42, 0.46) were separately synthesized. For Yb_0.25_Co_4_Sb_12_, stoichiometric amounts of Co powder (99.5%), Sb shot (99.99%), and Yb ingot (99.9%) were mixed and sealed in evacuated quartz tubes, which were slowly heated to 1323 K and held for 24 h, then cooled to 923 K and held for another 4 days, before furnace-cooled to room temperature. For (Ag_2_Te)_x_(Sb_2_Te_3_)_1−x_, appropriate amounts of Ag powder (99.9%), Sb shot (99.99%), and Te powder (99.99%) were mixed and sealed in evacuated quartz tubes, heated to 1273 K and held for 10 h, after that cooled to 923 K and then quenched in liquid-nitrogen. To obtain Yb_0.25_Co_4_Sb_12_-4 wt% (Ag_2_Te)_x_(Sb_2_Te_3_)_1−x_ composites (which will be denoted by matrix-100x hereafter), the Yb_0.25_Co_4_Sb_12_ ingot was pulverized manually in an agate mortar, and the (Ag_2_Te)_x_(Sb_2_Te_3_)_1−x_ ingot was milled in a planetary mill at 400 rmp for 5 h in vacuum. The Yb_0.25_Co_4_Sb_12_ coarse grains and (Ag_2_Te)_x_(Sb_2_Te_3_)_1−x_ particles were then mixed in a planetary mill at 300 rpm for 40 min in vacuum, and finally hot pressed into circular pellets at 873 K and 100 MPa for 2 h in argon atmosphere.

Phase characterization was performed by powder X-ray diffraction (PANalytical X'pert PRO diffractometer with Cu *Kα* radiation). Scanning electron microscopy (FEI: Quanta 200, FEI: Sirion 200), and field–emission transmission electron microscopy (FEI: Tecnai G2 F30) equipped with energy-dispersive X-ray spectroscopy (EDS) were used to inspect the microstructure. For high temperature thermoelectric property measurements, the samples were cut by diamond saw into 8 × 8 × 2 mm^3^ square for the thermal conductivity measurement and 12 × 2 × 3 mm^3^ bar for the electrical resistivity and Seebeck coefficient measurements. All the TE property measurements were carried out from 300 to 773 K. The Seebeck coefficient-electrical resistivity measurements were performed simultaneously on an Ulvac-Riko ZEM-2 system. The thermal conductivity κ was calculated via the relation κ *= DCd*, where the thermal diffusivity *D*, specific heat *C* were measured on a laser-flash apparatus (Shinkuriko: TC-7000H) in vacuum. The density was obtained from the measured weight and dimensions. Room temperature Hall coefficient measurement was performed on a Hall effect measurement system (Ecopia: HMS 5500) via Van der Pauw method under an applied magnetic field of 0.55 T. The carrier concentration *n* and Hall mobility μ_*H*_ were estimated from the measured Hall coefficient *R*_*H*_ and the electrical resistivity ρ via the relations, *n* = 1/*eR*_*H*_ and μ_*H*_ = *R*_*H*_/ρ, where *e* is the electron charge.

## Results and discussion

**Figure 1:**
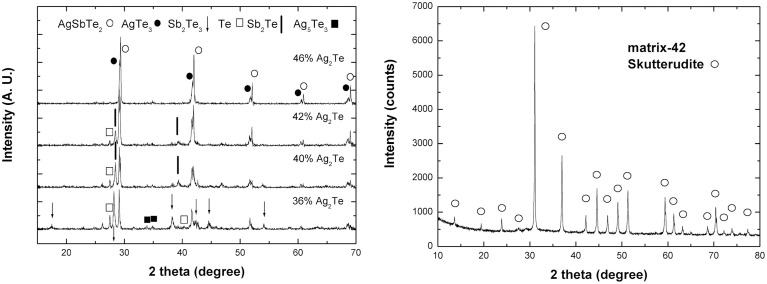
**(Left) The powder X-ray diffraction patterns of Ag_2x_Sb_2−2x_Te_3−2x_ ingots; (Right) The X-ray diffraction pattern of hot-pressed matrix-42 composite**.

The powder X-ray diffraction pattern of Yb_0.25_Co_4_Sb_12_ agrees well with a skutterudite structure, with the calculated lattice parameter to be 9.0438(2) Å. The enlargement of the lattice parameter compared to CoSb_3_ (JCPDS: 03-065-3144, 9.0347 Å) is consistent with the Yb-filling scenario. The powder X-ray diffraction patterns of (Ag_2_Te)_x_(Sb_2_Te_3_)_1−x_ ingots are shown in the left panel of Figure 1. As known, there is some disagreement in the literature about the pseudo-binary Ag_2_Te-Sb_2_Te_3_ slice of the Ag-Sb-Te phase diagram (Maier, 1963; Marin et al., 1985; Matsushita et al., 2004), indicating the phase complexity of this Ag-Sb-Te system. According to the pseudo-binary phase diagram, proposed by Marin et al. (1985), single-phase rock-salt structured AgSbTe_2_ falls in the range of *x* = 0.42–0.45 in (Ag_2_Te)_x_(Sb_2_Te_3_)_1−x_; when *x* > 0.45 Ag_2_Te forms in addition to the rock salt phase; and when *x* < 0.42 Sb_2_Te_3_ forms in addition to the rock salt phase. What we observed in the present work is somewhat different: the sample *x* = 0.46 is composed of rock-salt structured AgSbTe_2_ (JCPDS: 00-015-0540), rhombohedral AgTe_3_ (JCPDS: 01-076-2328), and barely discernible Ag_5_Te_3_ (JCPDS: 01-086-1168). It should be noted that the characteristic peaks of AgTe_3_ are very close to those of AgSbTe_2_. With decreasing x, the peak intensity of AgSbTe_2_ decreases while the characteristic peaks of single elemental Te and Sb-Te compound emerge. In the work reported by Wang et al. on (Ag_2_Te)_x_(Sb_2_Te_3_)_1−x_ (*x* = 0.44-0.50) synthesized via mechanical alloying—spark plasma sintering process (Wang et al., 2008), Ag_5_Te_3_ emerged instead of Ag_2_Te in addition to the primary rock salt phase in (Ag_2_Te)_0.48_(Sb_2_Te_3_)_0.52_ and (Ag_2_Te)_0.46_(Sb_2_Te_3_)_0.54_. These results indicate that the phase components of ternary (Ag_2_Te)_x_(Sb_2_Te_3_)_1−x_ system intimately depend on the synthesis process. Also shown in the right panel of Figure 1 is the XRD pattern of hot-pressed matrix-42 composite, and the other composites show similar results. The pattern indicates only the host matrix structure and no second phases associated with (Ag_2_Te)_x_(Sb_2_Te_3_)_1−x_ are detected, which is ascribed to the low content as well as the peak broadening effect of nano-phases.

**Figure 2:**
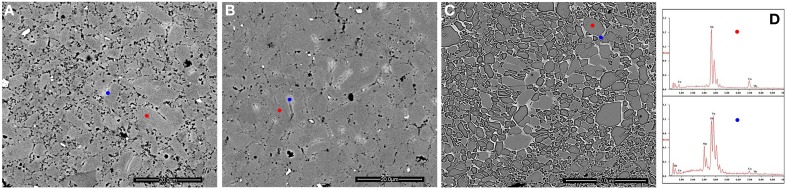
**(A)** BSE image of polished matrix-42 composite; **(B)** BSE image of polished matrix-40 composite; the red and blue points in **(A,B)** correspond to the host matrix and Ag-Sb-Te secondary phase respectively, as evidenced by EDS; **(C)** BSE image of polished and etched matrix-40 composite; **(D)** EDS results corresponding to the red and blue points in **(C)**.

**Figure 3:**
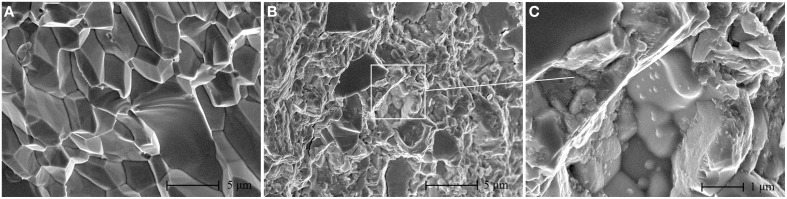
**The fractured surface SEM images of the studied composites: (A) Yb_0.25_Co_4_Sb_12_ matrix; (B) matrix-40; (C) a high-magnification image of the marked range in (B)**.

**Figure 4:**
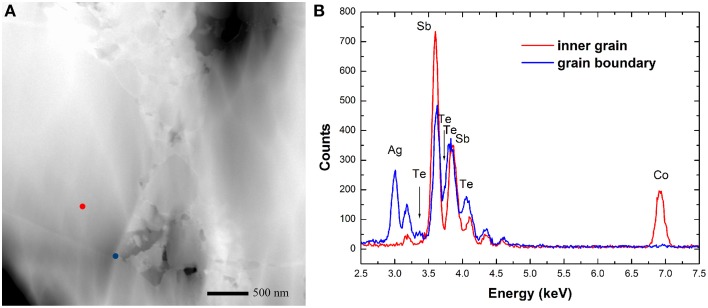
**(A)** Bright-field TEM image of sample matrix-42 and **(B)** the EDS results corresponding to the red and blue points in **(A)**.

Mechanically polished and fractured surfaces were both prepared for microstructural analysis. Figure 2 shows the back scattered electron (BSE) SEM images and EDS result of the polished samples. The secondary phases show different contrast with the matrix, as verified by EDS result, and are distributed on the grain boundaries of Yb_0.25_Co_4_Sb_12_ host matrix. The calculated average grain size of the host matrix by line transection method is 3 μm, consistent with the result obtained on the laser particle size analyzer. Figure 3 presents some representative fractured surface SEM images. The micro-morphology of the composites is different from that of the matrix especially on the grain boundary. A high-magnification image in Figure 3C shows nanoparticles with size of about 200 nm distributed on the grain boundaries. Furthermore, TEM observations and EDS analysis were performed on the sample matrix-42. No Ag or Te were detected inside the coarse grain but were rich on the grain boundaries (Figure 4).

**Figure 5:**
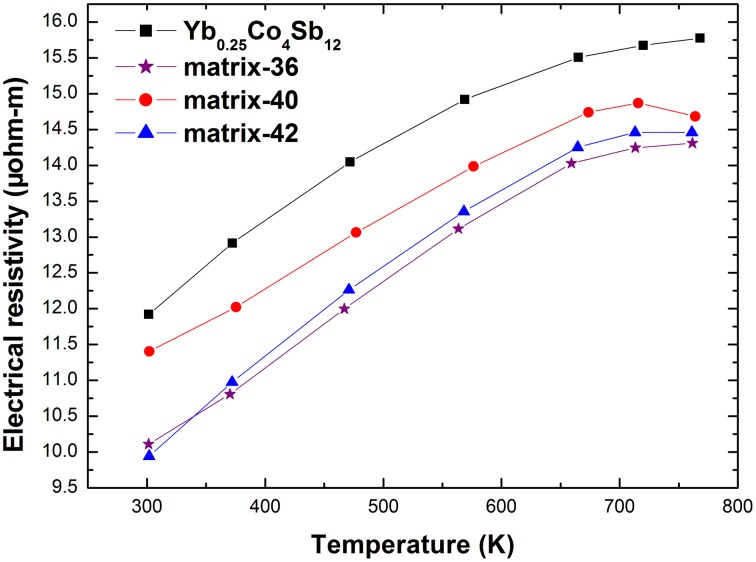
**High temperature electrical resistivity of the studied materials**.

**Figure 6:**
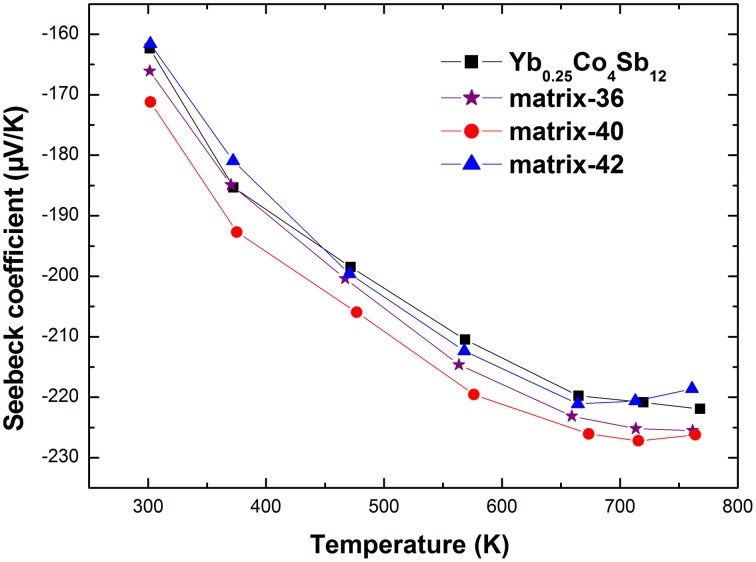
**High temperature Seebeck coefficient of the studied materials**.

Figures 5, 6 present the electrical resistivity and the Seebeck coefficient of the samples from 300 to 773 K, respectively. Consistent with the previous reports (Fu et al., 2013; Peng et al., 2014), the electrical resistivity of the composites was lowered while the absolute Seebeck coefficient was enhanced upon the addition of Ag-Sb-Te second phases, which is hard to achieve in conventional single-phased bulk material. The electrical resistivity of all materials increases with increasing temperature until it reaches a maximum, typical of heavily-doped semiconductor. Table 1 lists some room temperature physical properties of the samples studied. The carrier concentration, *n*, of the composites is slightly increased in relative to that of the pristine sample, presumably due to Te from the (Ag_2_Te)_x_(Sb_2_Te_3_)_1−x_ doping the Sb-site of the host matrix through diffusion in the hot pressing process. Notably, the carrier mobility μ_*H*_ is retained despite an increased *n*. This observation is somewhat unexpected because the carrier mobility is usually degraded upon nanocompositing since the interfaces scatter charge carriers (Clinger et al., 2012; Lee et al., 2012). In conjunction with the results of microstructure analysis, one possible scenario is that the Ag-Te-Sb second phases on the grain boundaries have improved the inter-grain electrical conductivity. Similar phenomena have been reported in Ba_0.3_Co_4_Sb_12_/Ag (Zhou et al., 2012) and also in our previously reported Yb_0.2_Co_4_Sb_12_-AgSbTe_2_ nanocomposites (Peng et al., 2014).

**Table 1:** **Some room temperature physical properties of the studied materials**.

**Composition**	***n* (10^20^/cm^3^)**	**ρ (μohm-m)**	**μ (cm^2^/Vs)**	**α (μV/K)**	**κ (W/m-K)**	**κ_*lattice*_ (W/m-K)**	m^*^(m_0_*)*	***mfp_electron_* (nm)**	***mfp_phonon_* (nm)**	***ZT***
Yb_0.25_Co_4_Sb_12_	−1.09	11.9	48.1	−162	2.84	2.34	1.9	4.3	1.5	0.23
Matrix-36	−1.28	10.1	48.3	−166	2.10	1.51	2.1	4.6	1.0	0.39
Matrix-40	−1.19	11.4	46.0	−171	2.18	1.65	2.1	4.4	1.1	0.35
Matrix-42	−1.24	9.9	50.8	−162	2.00	1.39	2.0	4.7	0.9	0.40

**Figure 7:**
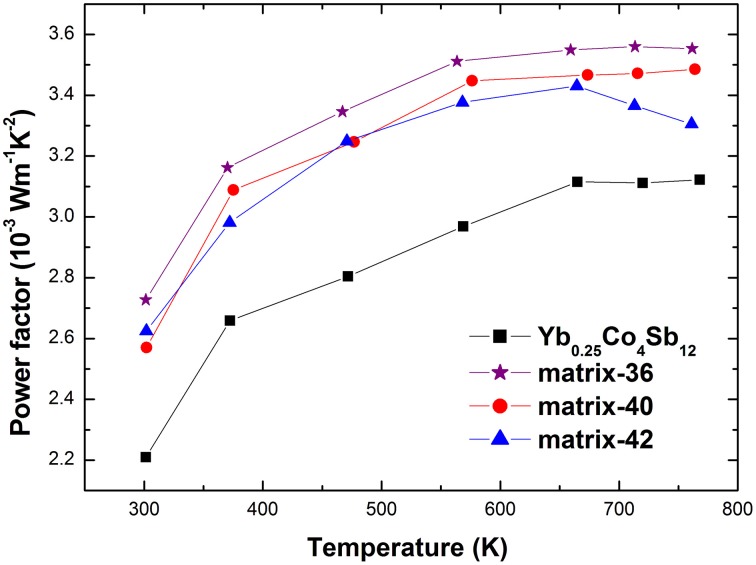
**High temperature power factor of the studied materials**.

Another interesting observation is the increased absolute Seebeck coefficient. For a series of samples that possess the same scattering mechanism and approximately the same effective mass, the absolute Seebeck coefficient is expected to be inversely correlated with the carrier concentration (Ganguly et al., 2011). However, as shown in Figure 6 the absolute Seebeck coefficient is increased compared with the pristine sample despite of an increased carrier concentration. Since, Ag-Sb-Te phases are distributed on the grain boundaries of the host matrix, energy filtering mechanism is expected to contribute to the enhanced Seebeck coefficient which preferentially scatters the low energy charge carriers (Xu et al., 2010). In addition, the increment is less than in the Yb_0.2_Co_4_Sb_12_-AgSbTe_2_ system (Peng et al., 2014). For example, the absolute Seebeck coefficient of matrix-40 is increased to 171 μV/K as compared to 162 μV/K of Yb_0.25_Co_4_Sb_12_ at 300 K, while that of Yb_0.2_Co_4_Sb_12_-4wt% (Ag_2_Te)_0.5_(Sb_2_Te_3_)_0.5_ is increased to 202 μV/K as compared to 152 μV/K of Yb_0.2_Co_4_Sb_12_ (Peng et al., 2014). This is ascribed to the different structure component of Ag-Sb-Te inclusion due to Ag/Sb ratio variation. Considering the structure component of Ag-Sb-Te inclusion is different from the pseudo-binary Ag_2_Te-Sb_2_Te_3_ phase diagram, further investigation of the synthesis process may be required to obtain the phase diagram consistent structure component. The lowered electrical resistivity in addition to the increased absolute Seebeck coefficient gives rise to an enhancement of the power factor (Figure 7).

**Figure 8:**
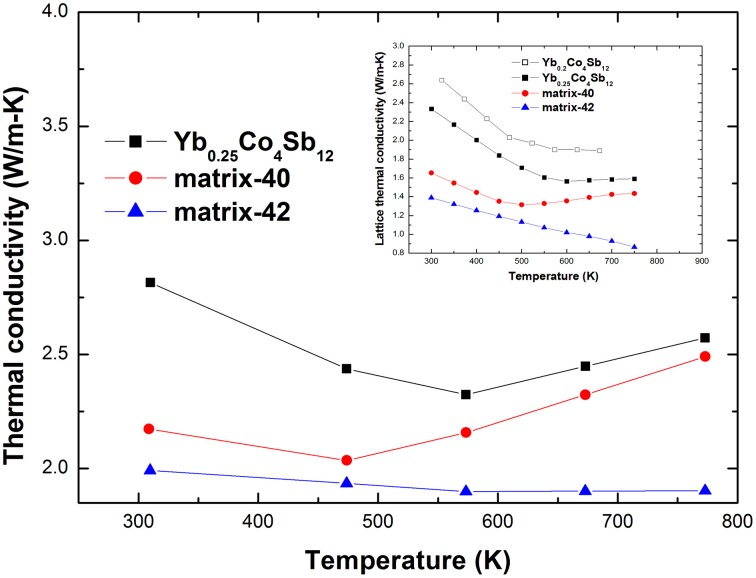
**High temperature thermal conductivity and lattice thermal conductivity of the studied materials, the lattice thermal conductivity of Yb_0.2_Co_4_Sb_12_ from literature (Fu et al., 2013) is present for comparison**.

Figure 8 presents the κ and κ_*L*_ of the studied materials from 300 to 773 K. The κ_*L*_ is estimated by subtracting the κ_*e*_ from the κ using the Wiedemann–Franz relation κ_*e*_ = *L*_0_*T*/ρ, with the Lorentz constant given by *L*_0_ = 2 × 10^−8^ V^2^K^−2^, which is suitable for heavily doped semiconductors (Goldsmid, 1986). The κ and κ_*L*_ were decreased systematically upon the addition of Ag-Sb-Te inclusions, and the κ_*L*_ decrease was more significant than that of Yb_0.2_Co_4_Sb_12_-AgSbTe_2_ system (Peng et al., 2014). For example, κ_*L*_ of matrix-42 shows a 39% decrease compared with Yb_0.25_Co_4_Sb_12_ matrix at 300 K, while that of Yb_0.2_Co_4_Sb_12_-4wt% (Ag_2_Te)_0.5_(Sb_2_Te_3_)_0.5_ shows a 27% decrease compared with Yb_0.2_Co_4_Sb_12_ matrix (Peng et al., 2014). Since, the detailed interactions between heat-carrying phonons and interfaces can be complex and material dependent (Cahill et al., 2003; Medlin and Snyder, 2009), it appears that perhaps the most important material parameter underlying the lattice thermal conductivity reduction is the interfacial area per unit volume (interface density) (Dresselhaus et al., 2007). From the XRD results, it is known that the (Ag_2_Te)_x_(Sb_2_Te_3_)_1−x_ series have complicated phase components, which will generally increase phase interface density and then decrease the κ_*L*_. Similar phenomena had been observed in our previously investigated In_0.2+x_Co_4_Sb_12+x_ composites, where the high interface density via the formation of InSb/Sb eutectic mixture on the grain boundaries considerably decreased the κ_*L*_ (Peng et al., 2012b).

To help quantify the impact of Ag-Sb-Te nanoinclusions on the carrier mobility and the phonon scattering, the room temperature electron mean-free-path and phonon mean-free-path have been estimated. In degenerate semiconductors with a parabolic band and acoustic phonon scattering, the Seebeck coefficient (Jeffrey Snyder and Toberer, 2008), the carrier mobility and the electron mean-free-path can be expressed by:

(1) $$\alpha =\frac{8\pi^{2}\kappa_{B}^{2}}{3eh^{2}}m^{*}T{\left(\frac{\pi }{3n}\right)}^{2/3}$$

(2) $$\mu =\frac{e\tau_{c}}{m^{*}}$$

(3) $$\frac{1}{2}m^{*}{\bar{v_{th}}}^{2}=\frac{3}{2}\kappa_{B}T$$

(4) $$l_{n}=\bar{v_{th}}\;\tau_{c}=\sqrt{\frac{3\kappa_{B}T}{m^{*}}}•\frac{m^{*}\mu }{e}=\sqrt{\frac{3\kappa_{B}Tm^{*}\mu^{2}}{e^{2}}}$$

Where κ_*B*_, h, m^*^, τ_*c*_, *v_th_*, and *l*_*n*_ are the Boltzmann constant, the Planck constant, effective mass, relaxation time, average thermal velocity, and mean-free-path, respectively. Per Equation (1), the effective mass was estimated to be in the range of 1.9 to 2.1*m*_0_. Incorporating Equations (2) and (3) to (4), the electron mean-free-path was estimated and listed in Table 1. Furthermore, the phonon mean-free-path can be estimated by the following relation:

(5) $$\kappa_{l}=\frac{1}{3}C_{v}\upsilon_{s}l_{p}$$

Where *C*_*v*_, υ_*s*_, *l*_*p*_ are the constant volume heat capacity, sound velocity, and phonon mean-free-path, respectively. We choose υ_*s*_ = 3400*m*/*s* from literature (Sales et al., 1997). The calculated phonon mean-free-path is listed in Table 1. It can be seen that the phonon mean-free-path decreases substantially while the electron counterpart slightly increases. Though the theoretical analysis of thermal conductivity (electronic conductivity) is difficult for nanocomposite with varying-size inclusions, since the wave length and mean-free-path for phonons (or electrons) at certain energy are unknown for most materials system (Liu et al., 2012), it is necessary to note a fundamental difference between the electrical and heat transport. While those electrons near the Fermi level contribute to the electrical transport, phonon modes in the entire Brillouin zone generally contribute to the heat transport. As such, a multiple length scale microstructure is generally beneficial to effectively scatter heat-carrying phonons over a wider wavelength range and also over a wide temperature range, while imposing less influence on electron transport. Even, when the average wavelength of heat-carrying phonons is getting shorter at elevated temperatures, in which case the short-range defects are more effective in scattering phonons, the presence of long-range defects such as nanoinclusions and interfaces is indispensable to effectively suppress the lattice thermal conductivity. In this context, the microstructure characteristic of the multi-phase nanocomposites distributed on the grain boundaries of micro-grained matrix in the studied composites allows for a greater tunability of {ρ, α, κ} as a group.

**Figure 9:**
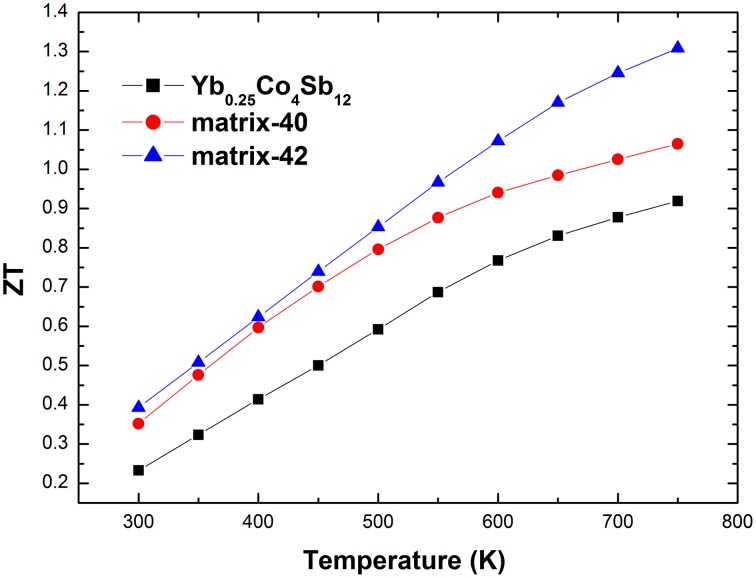
**High temperature dimensionless figure of merit *ZT* of the studied materials**.

Finally, Figure 9 presents the dimensionless figure of merit *ZT* of the studied materials from 300 to 773 K. The *ZT* values of the composites are enhanced in the entire temperature range, due to the simultaneous enhancement of the power factor and the reduction of the κ_*L*_. A maximum *ZT* of 1.3 was obtained in matrix-42 at 773 K, a 42% increase compared with that of the matrix.

## Conclusions

Yb_0.25_Co_4_Sb_12_-4wt% (Ag_2_Te)_x_(Sb_2_Te_3_)_1−x_ composites have been prepared via an *ex situ* melting-milling-hot-pressing process. The microstructure and high temperature thermoelectric properties have been investigated and correlated. Powder XRD analysis reveals the (Ag_2_Te)_x_(Sb_2_Te_3_)_1−x_ series have complicated phase components, which closely depend on the conditions of synthesis process. In the composites, the Ag-Sb-Te inclusions are distributed on the grain boundaries of Yb_0.25_Co_4_Sb_12_ coarse grains. Compared to pristine sample, the electrical conductivity and absolute Seebeck coefficient of the composites are enhanced simultaneously. In the meantime the thermal conductivity and the lattice thermal conductivity are lowered substantially upon the addition of Ag-Sb-Te inclusions. Hence we have present a case study that multi-phase nanocomposites and complicated nanostructures formed by varying Ag_2_Te/Sb_2_Te_3_ ratios within (Ag_2_Te)_x_(Sb_2_Te_3_)_1−x_ materials allows for a greater tunability of {ρ, α, κ} as a group. A maximum *ZT* of 1.3 was obtained in matrix-42 at 773 K, a 42% increase compared with the pristine sample. The present work reveals that the three thermoelectric parameters can be optimized simultaneously in CoSb_3_-based nanocomposites. Further investigation to clarify the underlying mechanisms is ongoing.

### Conflict of interest statement

The authors declare that the research was conducted in the absence of any commercial or financial relationships that could be construed as a potential conflict of interest.
